# Epidemiology and Risk Factors for Cryptosporidiosis in Children From 8 Low-income Sites: Results From the MAL-ED Study

**DOI:** 10.1093/cid/ciy355

**Published:** 2018-04-26

**Authors:** Poonum S Korpe, Cristian Valencia, Rashidul Haque, Mustafa Mahfuz, Monica McGrath, Eric Houpt, Margaret Kosek, Benjamin J J McCormick, Pablo Penataro Yori, Sudhir Babji, Gagandeep Kang, Dennis Lang, Michael Gottlieb, Amidou Samie, Pascal Bessong, A S G Faruque, Esto Mduma, Rosemary Nshama, Alexandre Havt, Ila F N Lima, Aldo A M Lima, Ladaporn Bodhidatta, Ashish Shreshtha, William A Petri, Tahmeed Ahmed, Priya Duggal

**Affiliations:** 1 Bloomberg School of Public Health, Johns Hopkins University, Baltimore, Maryland; 2 International Centre for Diarrhoeal Disease Research, Bangladesh, Dhaka; 3 Fogarty International Center, Bethesda, Maryland; 4 University of Virginia School of Medicine, Charlottesville; 5 Christian Medical College, Vellore, India; 6 Foundation for the National Institutes of Health, Bethesda, Maryland; 7 University of Venda, Thohoyandou, South Africa; 8 Haydom Global Health Institute, Tanzania; 9 Clinical Research Unit and Institute of Biomedicine, Universidade Federal do Ceara, Fortaleza, Brazil; 10 Armed Forces Research Institute of Medicine (AFRIMS), Bangkok, Thailand; 11 Walter Reed AFRIMS Research Unit Nepal, Kathmandu

**Keywords:** Cryptosporidium species, diarrhea, malnutrition, stunting, MAL-ED

## Abstract

**Background:**

*Cryptosporidium* species are enteric protozoa that cause significant morbidity and mortality in children worldwide. We characterized the epidemiology of *Cryptosporidium* in children from 8 resource-limited sites in Africa, Asia, and South America.

**Methods:**

Children were enrolled within 17 days of birth and followed twice weekly for 24 months. Diarrheal and monthly surveillance stool samples were tested for *Cryptosporidium* by enzyme-linked immunosorbent assay. Socioeconomic data were collected by survey, and anthropometry was measured monthly.

**Results:**

Sixty-five percent (962/1486) of children had a *Cryptosporidium* infection and 54% (802/1486) had at least 1 *Cryptosporidium*-associated diarrheal episode. *Cryptosporidium* diarrhea was more likely to be associated with dehydration (16.5% vs 8.3%, *P* < .01). Rates of *Cryptosporidium* diarrhea were highest in the Peru (10.9%) and Pakistan (9.2%) sites. In multivariable regression analysis, overcrowding at home was a significant risk factor for infection in the Bangladesh site (odds ratio, 2.3 [95% confidence interval {CI}, 1.2–4.6]). Multiple linear regression demonstrated a decreased length-for-age *z* score at 24 months in *Cryptosporidium*-positive children in the India (β = –.26 [95% CI, –.51 to –.01]) and Bangladesh (β = –.20 [95% CI, –.44 to .05]) sites.

**Conclusions:**

This multicountry cohort study confirmed the association of *Cryptosporidium* infection with stunting in 2 South Asian sites, highlighting the significance of cryptosporidiosis as a risk factor for poor growth. We observed that the rate, age of onset, and number of repeat infections varied per site; future interventions should be targeted per region to maximize success.

## Teaser

In this longitudinal multicenter study, 65% of children experienced *Cryptosporidium* infection during the first 2 years of life. *Cryptosporidium* was associated with severe diarrhea and dehydration and, in 2 South Asian sites, with stunted growth at age 2 years.

Diarrheal disease is a leading cause of death in children worldwide [1]. Cryptosporidiosis is a primary cause of moderate-to-severe diarrhea, and recent estimates suggest that annually *Cryptosporidium* species are responsible for >200000 deaths in children <2 years of age in South Asia and sub-Saharan Africa, and associated with morbidity in >7 million children in these regions [2, 3]. Despite its significant impact on early childhood morbidity and mortality, cryptosporidiosis remains without a vaccine, effective treatment, or environmental intervention.

*Cryptosporidium* species are enteric diarrheagenic protozoa that can cause fulminant infection in immunocompromised patients and children. *Cryptosporidium* infection has been associated with longer duration of diarrhea and 2–3 times higher mortality in children compared with age-matched children without diarrhea [3–5]. In addition to higher mortality, studies from Brazil and Peru have noted short-term growth faltering and impaired cognitive development after *Cryptosporidium* diarrhea [6–8]. Beyond diarrheal disease, subclinical carriage of the parasite has been associated with growth faltering [7, 9]. The relationship between malnutrition and *Cryptosporidium* infection is circuitous, as stunting has been identified as a risk factor and consequence of infection [10]. Other described risk factors for *Cryptosporidium* infection in children include poverty [9], overcrowding [11–14], contact with domesticated animals [15, 16], and exposure to human immunodeficiency virus–infected family members [17].

Previous studies have characterized the region-specific risk factors, but we lack a community-based multisite study on the epidemiology of cryptosporidiosis in young children. The Etiology, Risk Factors, and Interactions of Enteric Infections and Malnutrition and the Consequences for Child Health and Development Project (MAL-ED) identified *Cryptosporidium* as a common pathogen [18] and provided the opportunity to evaluate the epidemiology of cryptosporidiosis. MAL-ED followed children for the first 2 years of life across 8 sites in South America, sub-Saharan Africa, and Asia. We aimed to understand the epidemiology, risk factors, and clinical manifestations of *Cryptosporidium* within this longitudinal community-based study.

## METHODS

Enrollment occurred between November 2009 and February 2012 at 8 sites: Dhaka, Bangladesh (BGD); Fortaleza, Brazil (BRF); Vellore, India (INV); Bhaktapur, Nepal (NEB); Loreto, Peru (PEL); Naushero Feroze, Pakistan (PKN); Venda, South Africa (SAV); and Haydom, Tanzania (TZH) [19–26]. Children were enrolled within 17 days of birth and actively surveyed through 24 months. Ethical approval was obtained from all appropriate institutional review boards. Written informed consent was obtained from the parents. Details of the study design and microbiologic methods have been published [27, 28].

### Data Collection

At enrollment, household demographics were obtained by survey, and child birthdate and sex were recorded. Baseline child length and weight were measured and subsequently collected prospectively each month. Length-for-age and weight-for-age adjusted *z* scores (LAZ and WAZ, respectively) were calculated. Details of illness were collected during twice-weekly household visits throughout the study period [27].

### Sample Collection and Testing

Active surveillance for diarrhea was performed through interview of caregivers; during diarrheal illness, a diarrheal specimen was collected when possible. Diarrhea was defined as ≥3 loose stools per day, or at least 1 loose stool with blood; a new diarrheal episode was identified as being separated from the last by >2 diarrhea-free days [27]. Surveillance (nondiarrheal) stool specimens were collected monthly through 24 months of life; beyond year 1, only stools at months 15, 18, 21, and 24 were tested for enteropathogens. Stool specimens were collected, preserved, transported to the laboratories, and processed at all the sites using harmonized protocols. Testing for *Cryptosporidium* species was performed by a pan-*Cryptosporidium* immunoassay (TechLab, Blacksburg, Virginia). Methods of assessment of other enteropathogens were assessed using published methods [28]. All protocol-collected surveillance stools and the first diarrheal stool sample collected per diarrheal episode were included in the analysis. “Symptomatic infection” was defined as a diarrheal episode testing positive for *Cryptosporidium*, and “subclinical infection” was defined as a surveillance stool testing positive for *Cryptosporidium*. A new *Cryptosporidium* infection was defined as detection of *Cryptosporidium* in a diarrheal or surveillance stool with negative testing in the 30 days prior.

### Clinical and Socioeconomic Characteristics

Dehydration was categorized as “some” dehydration, with a child being thirsty, irritable, with sunken eyes, or reduced skin turgor, or “severe” dehydration including lethargy and listlessness [27]. Diarrhea severity was scored using the Global Enteric Multicenter Study (GEMS) severity score [3]. “Moderate-severe” diarrhea was associated with dehydration, dysentery, or hospitalization, and “mild” diarrhea denoted the absence of these 3 indicators.

Monthly income was converted to US dollars and log transformed. Mothers’ schooling was categorized as follows: no school, ≤5 years, and >5 years. “Overcrowding” in the home was classified as >3 people per room per household [29]. “Unimproved” drinking water was access only to surface water or unprotected well water as compared to “improved” drinking water, which included piped water, public tap, tube well, borehole, and protected well water [30]. “Unimproved” toilet was defined as having no facility, bucket toilet, or pit latrine without slab. “Improved” toilet included nonflush pit latrine with slab and flush toilet to piped sewer system, septic tank, or pit latrine [30]. “Unimproved” household flooring was composed of earth, sand, clay, mud, or dung. “Improved” flooring was made up of wood, ceramic tiles, vinyl, or concrete [31].

### Inclusion Criteria

Children were included in this analysis if they had anthropometry at baseline and at month 24. To avoid misclassification bias and be certain of *Cryptosporidium*-negative status, we further limited the analysis to children with complete stool testing for months 2–12 and labeled as *Cryptosporidium* negative, if their surveillance and diarrheal stools tested negative for *Cryptosporidium* during this period. Children with at least 1 *Cryptosporidium*-positive stool result during months 2–12 were included, and labeled as *Cryptosporidium* positive.

### Statistical Analysis

Demographic and clinical characteristics of included children were summarized based on socioeconomic factors and environmental risk factors for enteric infection.

Evaluation of symptoms and coinfections during *Cryptosporidium* diarrheal episodes was performed using *t* tests. Logistic regression was used to determine risk factors for *Cryptosporidium* infection, with infection categorized as a binomial response. Variables of interest, including family income, overcrowding, years of mother’s schooling, animal ownership, floor type, drinking water source, and toilet type were included if there was >5% heterogeneity per category per site. The significant heterogeneity in characteristics between sites required independent analysis of risk factors for each site. BRF was not included in the risk factor analysis because of the limited sample size and the lack of heterogeneity for most variables.

To evaluate whether preinfection nutritional status could be a risk factor for infection, the 3-month mean LAZ score preceding the time of infection was compared between infected and uninfected children using Welch’s 2-sample *t* test at 4 age groups: 3, 6, 9, and 12 months.

The association of *Cryptosporidium* (diarrheal and subclinical) infection during year 1 and growth (LAZ) at 24 months was performed using multiple linear regression. PKN was excluded from this growth analysis due to bias noted in the anthropometric results during quality control assessments. *Cryptosporidium* infection was categorized as a binary variable. Covariates included in the regression differed by site based on the level of heterogeneity in variables at that site (ie, BRF showed no heterogeneity in the other variables, so they could not be included). All sites included sex and baseline LAZ and the additional following variables were included per site: (BGD: income, overcrowding; INV: income, overcrowding, toilet type; NEB: income, chickens/ducks; PEL: toilet type, chickens/ducks; SAV: income, chickens/ducks, cattle; TZH: income, chickens/ducks, cattle; BRF no additional variables).

In a second analysis to evaluate linear relationship of *Cryptosporidium* infection in year 1 and 24-month LAZ, we applied inverse probability weighting to account for heterogeneity in variables across sites within a single model. Covariates included were site, sex, baseline LAZ, and the 6-month measurements of toilet type, water type, overcrowding, years of mother’s schooling, and family income.

Sequence plots were used to depict the *Cryptosporidium* shedding in stool over the follow-up period using the seqdef option of the TraMineR R-package. Kaplan-Meier survival analysis was used to visualize the time to first *Cryptosporidium* infection. All analyses were performed using Stata version 13 (StataCorp, College Station, Texas) and/or R version 3.2.2 (Foundation for Statistical Computing, Vienna, Austria) software.

## RESULTS

Of 2145 children enrolled in the MAL-ED study, 1659 children completed follow-up through 24 months, and of these, 1550 children had complete 12 months of stool testing available. A subset of these (n = 1486) had complete stool testing through age 2. Baseline LAZ in BRF was significantly higher than the other sites. Most households in INV, PEL, and TZH had unimproved sanitation facilities.

### Incidence of *Cryptosporidium* Infection

**Figure 1.**
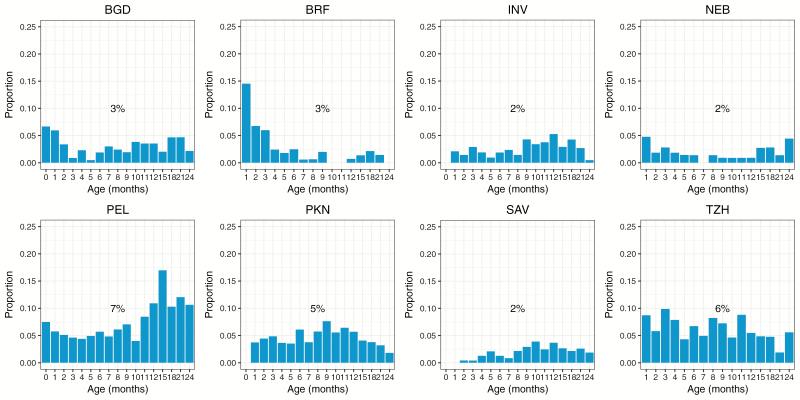
Percentage of surveillance stools positive for *Cryptosporidium* by age and by site. The first surveillance stool collected per child per month was included. Overall percentage of surveillance stools positive per site is summarized. Abbreviations: BGD, Dhaka, Bangladesh; BRF, Fortaleza, Brazil; INV, Vellore, India; NEB, Bhaktapur, Nepal; PEL, Loreto, Peru; PKN, Naushero Feroze, Pakistan; SAV, Venda, South Africa; TZH, Haydom, Tanzania.

During the 2-year follow-up period, 27418 surveillance stools were collected and tested from 1659 children, and 3.9% (1069) tested positive for *Cryptosporidium,* with the rate of positivity across sites ranging from 2% to 7% (Figure 1). PEL (7%) and TZH (6%) had the highest rates of subclinical infection.

**Figure 2.**
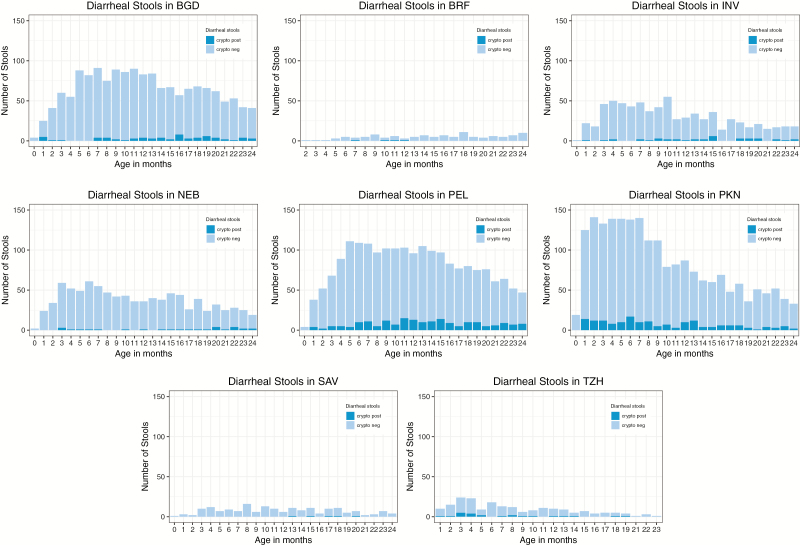
Number of diarrheal stools collected per month per site. The sites in Peru, Pakistan, and Bangladesh had the highest incidence of diarrhea, and the sites in Peru and Pakistan had the highest rates of *Cryptosporidium–*positive diarrheal stools. Abbreviations: BGD, Dhaka, Bangladesh; BRF, Fortaleza, Brazil; INV, Vellore, India; NEB, Bhaktapur, Nepal; PEL, Loreto, Peru; PKN, Naushero Feroze, Pakistan; SAV, Venda, South Africa; TZH, Haydom, Tanzania.

From these 1659 children, 7821 diarrheal stools were collected, of which 6.9% tested positive for *Cryptosporidium*. The incidence of diarrhea varied greatly between sites, with PEL and PKN having the highest incidence of diarrhea overall and of diarrheal episodes positive for *Cryptosporidium* (Figure 2). TZH, SAV, and BRF had a low incidence of diarrhea regardless of age. Within each site, the rate of diarrhea was constant over the first 2 years of life, except in PKN where diarrheal incidence peaked before 10 months of age but remained high through 24 months.

**Figure 3.**
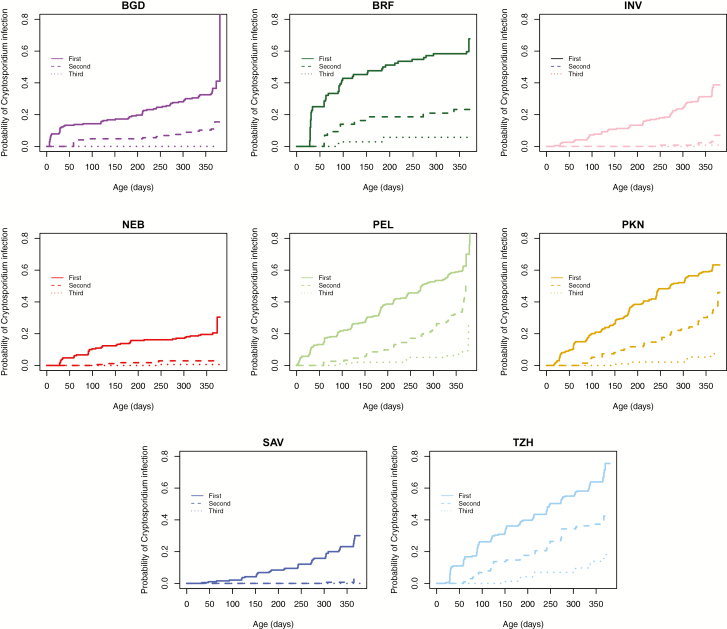
Time to Nth *Cryptosporidium* detection over the first year of life. This includes both diarrheal and asymptomatic *Cryptosporidium* infection. Children at the Peru, Pakistan, and Tanzania sites frequently had up to 3 infections with *Cryptosporidium*. Repeat infection was less commonly observed in the other 5 sites. Abbreviations: BGD, Dhaka, Bangladesh; BRF, Fortaleza, Brazil; INV, Vellore, India; NEB, Bhaktapur, Nepal; PEL, Loreto, Peru; PKN, Naushero Feroze, Pakistan; SAV, Venda, South Africa; TZH, Haydom, Tanzania.

Across MAL-ED, 65% (962/1486) of children had at least 1 *Cryptosporidium* infection and 54% (802/1486) had at least 1 *Cryptosporidium* diarrheal episode during the first 2 years of life. By site, NEB had the lowest (21%), and PEL (62%) and TZH (68%) the highest, percentage of children with cryptosporidiosis (Table 1). BRF, TZH, PKN, and PEL were the sites with fastest progression to first infection, and all had a median time to first infection before age 1 year (Table 2). Sites varied in terms of whether diarrheal or subclinical infection occurred first. For example, in PKN, time to first diarrheal infection was earlier than subclinical. Conversely, subclinical infection occurred earlier than diarrheal infection in BGD, BRF, NEB, and PEL; and in INV and TZH, both types of infection occurred around the same age. The rate of repeat infections in year 1 varied, with PKN, PEL, and TZH having greatest repeat infections, in contrast to INV and SAV, where repeat infections during year 1 were rare (Figure 3).

**Table 1.** Characteristics of Children With Complete Follow-up, by Site

Characteristic	BGD	BRF	INV	NEB	PEL	PKN	SAV	TZH
Children included per site, No.	203	84	195	210	243	234	190	191
*Cryptosporidium* positive, %	37	61	36	21	63	62	27	68
Female sex, %	49	51	53	48	45	50	47	52
Enrollment LAZ, mean (SD)	–1.0 (1.1)	–0.1 (1.2)	–1.0 (1.1)	–0.65 (1.0)	–1.3 (1.0)	–1.1 (1.2)	–0.84 (1.1)	–1.0 (1.1)
Exclusive breastfeeding days, median (IQR)	155 (117–176)	112 (64–152)	107 (75–138)	86 (43–131)	84 (29–133)	14 (8–20)	31 (19–52)	55 (35–79)
Household monthly income, USD, median (IQR)	108 (79–144)	348 (308–390)	61 (44–96)	138 (101–211)	127 (104–170)	127 (81–220)	192 (116–291)	15 (8–30)
Mother’s years of schooling, median (IQR)	5 (2–8)	9 (7–12)	8 (4–10)	9 (6–10)	8 (6–10)	0 (0–5)	11 (9–12)	7 (3–7)
Overcrowding, %	47	0	46	12	11	55	7	16.5
Poor sanitation, %	0	0	56	1	76	23	4	87
Unprotected water source, %	0	0	0	0	10	0	12	68
Cattle ownership, %	2	0	4	3	0	64	16	66
Chicken or duck ownership, %	7	6	10	34	40	50	39	88
Dirt floor, %	5	1	8	54	74	73	12	92

Abbreviations: BGD, Dhaka, Bangladesh; BRF, Fortaleza, Brazil; INV, Vellore, India; IQR, interquartile range; LAZ, length-for-age *z* score; NEB, Bhaktapur, Nepal; PEL, Loreto, Peru; PKN, Naushero Feroze, Pakistan; SAV, Venda, South Africa; SD, standard deviation; TZH, Haydom, Tanzania; USD, US dollars.

**Table 2.** Median Time to First *Cryptosporidium* Infection, by Site

Site	Median Time to First *Cryptosporidium* Infection			Median Time to First *Cryptosporidium* Diarrheal Episode			Median Time to First *Cryptosporidium* Subclinical Detection		
	Median Days (SD)	Number of Subjects	Number of Events	Median Days (SD)	Number of Subjects	Number of Events	Median Days (SD)	Number of Subjects	Number of Events
BGD	381 (126)	203	74	235 (113)	17	17	124 (134)	57	57
BRF	188 (149)	84	51	294 (77)	3	3	61 (81)	48	48
INV	…	195	71	272 (102)	14	14	248 (110)	57	57
NEB	…	210	44	161 (103)	9	9	93 (113)	35	35
PEL	274 (132)	243	154	234 (102)	48	48	123 (112)	106	106
PKN	272 (125)	243	146	139 (93)	69	69	212 (106)	77	77
SAV	…	190	51	…	…	…	273 (94)	51	51
TZH	249 (129)	191	130	143 (105)	8	8	153 (112)	122	122

The first column shows median time to first *Cryptosporidium* infection, including both diarrheal and subclinical infections. Only data from year 1 of life were included, due to incomplete testing for *Cryptosporidium* year 2 surveillance stools. INV, NEB, and SAV all had median time to first infection beyond 1 year, so median times could not be calculated using this model. The second column shows median time to first diarrheal infection. The third column shows median time to first subclinical infection. For SAV, there were no *Cryptosporidium* diarrheal events during this time period.

Abbreviations: BGD, Dhaka, Bangladesh; BRF, Fortaleza, Brazil; INV, Vellore, India; NEB, Bhaktapur, Nepal; PEL, Loreto, Peru; PKN, Naushero Feroze, Pakistan; SAV, Venda, South Africa; SD, standard deviation; TZH, Haydom, Tanzania.

### Clinical Characteristics

Across sites, episodes of *Cryptosporidium*-associated diarrhea were clinically associated with “some” dehydration in all age strata except the 6- to 12-month group, where non-*Cryptosporidium* diarrheal episodes were just as likely to be associated with dehydration (Table 3); notably, there were few “severe” dehydration symptoms associated with diarrhea in this study. In children <6 months of age, *Cryptosporidium* diarrhea was significantly associated with a higher diarrhea severity score based on the GEMS definition [32]. *Cryptosporidium*-positive diarrheal episodes were not associated with fever or bloody stool (data not shown).

**Table 3.** Clinical Characteristics and Coinfection in *Cryptosporidium*-Associated Diarrheal Episodes Compared to Non-*Cryptosporidium* Diarrheal Episodes, Stratified by Age

Characteristic	<6 mo			6–11 mo			12–17 mo			18–24 mo		
	–	+	*P* Value	–	+	*P* Value	–	+	*P* Value	–	+	*P* Value
No.	2189	133	…	2228	151	…	1696	146	…	1168	110	…
Vomiting, %	16.0	21.8	.10	18.2	17.9	1.0	14.2	8.2	.06	8.2	10.1	.62
Fever, %	3.3	3.0	.67	6.3	7.3	.35	4.7	4.8	.99	4.5	4.5	1.0
Days of diarrhea (mean)	5.74 (6.29)	5.98 (5.28)	.66	4.82	5.31	.21	4.31 (3.32)	4.92 (3.38)	.034	4.00 (3.41)	3.73 (2.54)	.41
Dehydration, %			**.005**			.22			**.047**			**.002**
None	91.5	83.5	…	89.2	84.8	…	89.5	84.2	…	90.9	90.0	…
Some	8.3	16.5	…	10.7	15.2	…	10.5	15.8	…	9.1	10.0	…
Severe	0.1	0.0	…	0.0	0.0	…	0.0	0.0	…	0.0	0.0	…
Moderate-severe diarrhea (GEMS definition), %	11.9	20.3	**.007**	15.4	19.2	.26	14.3	17.8	.98	14.6	12.7	.70
Diarrhea >14 days, %	6.7	8.3	.59	3.2	4.0	.80	1.9	2.1	1.0	1.4	0.9	1.0
Presence of other pathogen, %												
Any bacteria	51.8	52.7	.92	69.6	69.5	1.0	71.1	71.8	.93	74.7	70.5	.41
Any virus	25.0	28.6	.42	42.0	37.1	.27	36.7	29.5	.10	30.7	23.6	.15
Adenovirus	2.5	1.5	.66	5.2	2.6	.23	4.8	1.4	.09	4.8	3.6	.75
Astrovirus	4.4	4.5	1.0	5.8	4.6	.67	7.2	4.8	.36	4.8	3.6	.75
Rotavirus	4.1	4.5	1.0	7.8	2.0	**.013**	6.6	3.4	.19	6.2	3.6	.39
*Shigella*	0.2	0.8	.41	1.2	0.7	.87	4.0	2.8	.61	7.0	5.5	.71
*Campylobacter*	24.	32.8	.056	44.0	46.4	.64	46.7	47.9	.83	48.0	47.3	.96
*Giardia*	6.7	10.5	.13	15.6	23.2	**.019**	24.8	23.3	.77	35.6	32.7	.62
EAEC	25.7	22.9	.54	26.9	21.2	.15	21.0	19.3	.72	17.3	23.3	.16
	0–6 mo			6–12 mo			12–18 mo			18–24 mo		
Negative for all pathogens except *Cryptosporidium,* No.	55			29			22			17		

Significant *P*-values listed in bold.

Abbreviations: –, non-*Cryptosporidium* diarrheal episode; +, *Cryptosporidium*-associated diarrheal episode; EAEC, enteroaggregative *Escherichia coli*; GEMS, Global Enteric Multicenter Study.

### Copathogens

Among those children with a symptomatic *Cryptosporidium* infection in the first 6 months of life, one-third had a coinfection with *Campylobacter*, which was slightly higher than among those with no *Cryptosporidium* (32.8% vs 24.9%, *P* = .06). Conversely, in months 6–12, *Cryptosporidium*-negative diarrheal episodes were more likely to test positive for rotavirus (7.8% vs 2%, *P* = .01). No co-segregation was seen between *Cryptosporidium* diarrhea and other diarrheagenic pathogens including enteroaggregative *Escherichia coli*, *Shigella*, and adenovirus.

### *Cryptosporidium* Risk Factors

Table 4 summarizes the risk factor analysis per site. In both univariate and multivariate regression analysis, overcrowding was identified as a risk factor for *Cryptosporidium* infection (both subclinical and diarrheal), though only significant in BGD (univariate odds ratio [OR], 2.1 [95% confidence interval {CI}, 1.1–3.9]; multivariate OR, 2.33 [95% CI, 1.2–4.6]) (Table 4 and Supplementary Table 1). Children with a lower preceding mean 3-month LAZ were more likely to have a *Cryptosporidium* infection (6-month: LAZ –1.0 vs –1.2, *P* = .06; 9-month: LAZ –1.3 vs –1.1, *P* = .05; 12-month: LAZ –1.6 vs –1.3, *P* = .007) (Supplementary Table 2).

**Table 4.** Univariate Logistic Regression of Risk Factors for All *Cryptosporidium* Infections, Both Diarrheal and Subclinical, During the First 24 Months of Life per Site

Risk Factor		Odds Ratio (95% CI)					
	BGD	INV	NEB	PKN	PEL	SAV	TZH
Overcrowding	2.1 (1.1–3.9)	1.24 (.71–2.19)	1.35 (.54–3.35)	1.1 (.60–1.9)	1.1 (.31–3.9)	3.5 (.87–14.28)	0.85 (.29–2.48)
Dirt floor	1.8 (.46–6.7)	0.8 (.27–2.3)	1.14 (.64–2.0)	1.55 (.83–2.88)	1.5 (.66–3.34)	2.7 (.87–8.4)	0.28 (.04–2.18)
Poor sanitation	…	1.50 (.85–2.64)	…	1.3 (.64–2.69)	0.94 (.38–2.3)	…	0.2 (.03–1.55)
Unprotected water source	…	…	…	…	2.5 (.32–19.9)	2.30 (.77–6.89)	0.92 (.39–2.18)
Chickens or ducks kept in home	6.7 (.85–53.5)	1.14 (.45–2.89)	1.53 (.84–2.80)	0.68 (.38–1.22)	1.12 (.50–2.50)	0.72 (.35–1.48)	0.49 (.11–2.25)
Cattle kept in home	…	…	…	0.76 (.41–1.40)	…	0.35 (.12–1.02)	0.91 (.37–2.23)
Maternal schooling 1–5 y	0.86 (.38–1.97)	0.68 (.25–1.86)	0.83 (.25–2.78)	0.80 (.41–1.53)	…	…	0.84 (.26–2.8)
Maternal schooling >5 y	1.5 (.65–3.67)	0.59 (.24–1.45)	0.53 (.18–1.55)	0.70 (.32–1.53)	…	…	1.71 (.59–4.95)
Household income (log)	1.05 (.60–1.85)	1.47 (.90–2.4)	0.81 (.51–1.28)	0.90 (.60–1.36)	1.3 (.78–2.1)	0.76 (.47–1.22)	1.90 (1.15–3.12)

Variables with <5% heterogeneity between subcategories in this site were not included in analysis due to lack of power (indicated by ellipses).

Abbreviations: BGD, Dhaka, Bangladesh; BRF, Fortaleza, Brazil; CI, confidence interval; INV, Vellore, India; NEB, Bhaktapur, Nepal; PEL, Loreto, Peru; PKN, Naushero Feroze, Pakistan; SAV, Venda, South Africa; TZH, Haydom, Tanzania.

### *Cryptosporidium* Infection as Predictor of Growth

**Figure 4.**
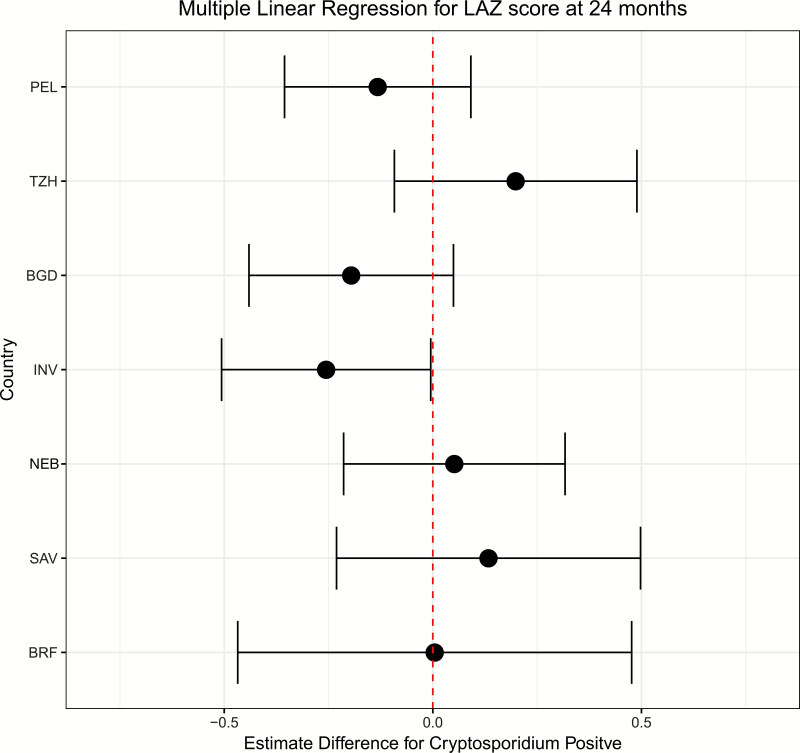
Forest plot depicting estimates of multiple linear regression of length-for-age *z* score at 24 months and *Cryptosporidium* infection during first 12 months of life, by site. Sites are ordered by burden of *Cryptosporidium* infection, with the Peru site having the most infections, and the Brazil site having the fewest. Abbreviations: BGD, Dhaka, Bangladesh; BRF, Fortaleza, Brazil; INV, Vellore, India; LAZ, length-for-age *z* score; NEB, Bhaktapur, Nepal; PEL, Loreto, Peru; PKN, Naushero Feroze, Pakistan; SAV, Venda, South Africa; TZH, Haydom, Tanzania.

We evaluated the relationship between *Cryptosporidium* infection during the first year of life and its impact on LAZ at 24 months using linear regression for each of 7 sites (PKN excluded, 1328 children). In 2 South Asian sites, INV and BGD, children with a *Cryptosporidium* infection during year 1 had a 0.25 lower LAZ at 24 months (INV: β = –.26 [95% CI, –.51 to –.01]; BGD: β = –.25 [95% CI, –.49 to –.01]) compared to children without a *Cryptosporidium* infection during year 1 (Table 5), but this association was not seen in the other sites (Figure 4). A similar trend was noted in INV and BGD for LAZ at 12 and 18 months (Supplementary Table 3). Linear regression of *Cryptosporidium* infection on the 24-month LAZ across sites using inverse probability weighting to accommodate multiple risk factors gave similar results (β = –.08 [95% CI, –.22 to .06]).

**Table 5.** Linear Regression of Association of *Cryptosporidium* Infection (Includes Both Diarrheal and Subclinical) in First 12 Months of Life and Length-for Age *z* Score at 24 Months

Site	No.	β (95% CI)
BGD	172	–.20 (–.44 to .05)
BRF	70	.00 (–.47 to .48)
INV	187	–.26 (–.51 to –.01)
NEB	204	.06 (–.20 to .32)
PEL	186	–.13 (–.36 to .09)
SAV	128	.13 (–.23 to .49)
TZH	165	.19 (–.09 to .48)

Total No. of children included was 1328 (222 children from Pakistan were excluded).

Abbreviations: BGD, Dhaka, Bangladesh; BRF, Fortaleza, Brazil; CI, confidence interval; INV, Vellore, India; NEB, Bhaktapur, Nepal; PEL, Loreto, Peru; PKN, Naushero Feroze, Pakistan; SAV, Venda, South Africa; TZH, Haydom, Tanzania.

## DISCUSSION

This is the first multicountry prospective cohort study of clinical and predictive risk factors for *Cryptosporidium* infection in community-dwelling children using harmonized protocols across 8 sites. This study provides incidence of diarrheal and subclinical *Cryptosporidium* infection in children from birth to 2 years of age from sites in South Asia, sub-Saharan Africa, and South America.

Our study of 1486 children demonstrated that *Cryptosporidium* is a common pathogen, affecting 65% of subjects, and is associated with diarrheal illness in 54%. In all sites, *Cryptosporidium* infection in the first 6 months of life was associated with more severe illness as measured by the GEMS severity score. The primary driver of severity was likely dehydration. Other clinical signs, including fever, dysentery, and vomiting, were not associated with *Cryptosporidium* diarrhea. And although severe dehydration was rare in this community-based study, *Cryptosporidium* diarrheal episodes were more likely associated with some dehydration vs non-*Cryptosporidium* diarrheal episodes. These findings suggest that younger children are more susceptible to severe *Cryptosporidium* disease, and that community-based programs for oral rehydration may play an important role in limiting morbidity from *Cryptosporidium*, as with other diarrheal pathogens.

In addition to diarrhea-associated infections, ours is the first longitudinal study to report rates of subclinical *Cryptosporidium* infection in 8 sites. We found that Loreto, Peru (7%) and Haydom, Tanzania (6%) were sites with highest rates of subclinical *Cryptosporidium* infection, which is significant as the burden of subclinical infections in these locations has not been previously reported at this scale. Also of note, in Fortaleza, Brazil, though children had a better nutritional status at enrollment, and less diarrhea, we found the incidence of subclinical *Cryptosporidium* infection to be 3%, suggesting that subclinical infection is an important problem at this site. The incidence of subclinical infections in South Asia was lower than previously reported [9, 33, 34]. This difference likely reflects differences in testing. For the current study, we used antigen detection for *Cryptosporidium* as compared to the use of the higher-sensitivity quantitative polymerase chain reaction in the prior Indian and Bangladeshi studies [9, 32, 33]. A recent reanalysis of the GEMS study has shown that enzyme-linked immunosorbent assay (ELISA) may be just as sensitive as molecular diagnostics for *Cryptosporidium* in diarrheal disease [35]. However, as nondiarrheal subclinical infection carries a lower parasite burden, the ELISA may have underestimated incidence of subclinical infection in MAL-ED, and more sensitive testing is warranted.

Overcrowding, lower 3-month growth velocity, poor sanitation, and poultry were associated with increased risk of infection. Overcrowding is a known risk factor for cryptosporidiosis [11–14] and may be a corollary for lower socioeconomic status; alternatively, overcrowding in the home may increase risk of person-to-person transmission between household members. We did not find an association of cryptosporidiosis with unprotected drinking water, supporting results of an Indian study that demonstrated no reduced risk of cryptosporidiosis with drinking bottled water vs water from the municipal supply [36]. This suggests that other factors, including crowding, poor sanitation, and high environmental burden, may promote transmission of infection to young children.

This study demonstrated a significant association of *Cryptosporidium* infection during year 1 and linear growth faltering at 24 months of age in INV and a trend toward significance in BDG, consistent with a prior study from this region [9]. In the other sites, no significant relationship between infection and LAZ score at 24 months was observed. There are several potential reasons for these divergent findings. Our model did not account for nutritional intake as a component of growth, though other MAL-ED publications have evaluated this and found no significant signal [37]. In addition, the epidemiology and prevalence of different *Cryptosporidium* species and subspecies has not been extensively described, and may differ greatly across the sites we studied. Molecular studies have suggested differences in clinical presentation between *Cryptosporidium* species; for example, *Cryptosporidium hominis* has been associated with more severe dehydration, and *Cryptosporidium meleagridis* with mild disease [6, 37, 38]. Thus, different species may also vary in pathogenic impact on child growth. It is also possible that there is residual confounding of unknown risk factors.

A novel finding from this study is the variability in the age of onset of *Cryptosporidium* infection. In BRF, PEL, PKN, and TZH the median age at first infection was in the first year of life whereas in the other 4 sites, the median age was >1 year. The finding from BGD is consistent with a prior report from Bangladesh, which reported median age at first infection to be 13.9 months [9]. In the sites with earliest age of onset, there was also the greatest rate of repeat infections. Earlier onset of infection and greater probability of repeat infection could be due to greater burden of circulating parasite in that environment. This could also be attributed to failure of host immune response, either related to host genetics or lack of protection from maternal breast milk antibodies [32, 39]. It is also possible that infection by one *Cryptosporidium* species does not afford protection from all other species, and in sites with repeat infections there is greater genotypic diversity of the parasite, increasing risk of recurrent infection. Further studies are needed to understand whether acquired immunity to *Cryptosporidium* is species or genotype specific.

This study had limitations. Our analysis was limited to including infections in year 1 of life, due to incomplete testing in year 2 per the study design, and did not account for the impact of a large burden of infections that occurred in the second year of life. Although we have details on some of these infections, we lacked complete follow-up so could not categorize someone as not having an infection, and similarly a child may have had an infection and resolved it.

Furthermore, heterogeneity in multiple factors between sites, as well as limited sample sizes within sites, significantly impaired ability to draw cross-site and per-site conclusions. Last, we were unable to test infection in household members and environmental samples to more accurately describe exposure risk.

MAL-ED represents the largest multicountry longitudinal investigation of *Cryptosporidium* infection in children. Our study confirmed that *Cryptosporidium* infection is a significant contributor to diarrhea morbidity in community-dwelling children, and found that a majority of children across sites experienced infection before age 2. The differences in age of onset, diarrhea-associated or subclinical infection, and the rate of repeat infections across sites indicates that site-specific characteristics must be considered when designing infection control strategies for *Cryptosporidium.* Given our findings of associations between *Cryptosporidium* and short- and long-term morbidity, efforts at prevention and control of the parasite should focus on areas that have seen high rates of infection in the first year of life (PEL, PKN, TZH) and an association between *Cryptosporidium* infection and malnutrition (BGD and INV).

## Supplementary Data

Supplementary materials are available at *Clinical Infectious Diseases* online. Consisting of data provided by the authors to benefit the reader, the posted materials are not copyedited and are the sole responsibility of the authors, so questions or comments should be addressed to the corresponding author.

Supplemental_Table_1

Supplemental_Table_2

Supplemental_Table_3

Supplemental_Figure_1_Page_1

Supplemental_Figure_1_Page_2

Supplemental_Figure_1_Page_3

Supplemental_Figure_1_Page_4

Supplemental_Figure_1_Page_5

Supplemental_Figure_1_Page_6

Supplemental_Figure_1_Page_7

Supplemental_Figure_1_Page_8

Supplemental_Figure_legend

## Notes

***Acknowledgments.*** We thank the children and families of the MAL-ED network for their participation in this study.

***Financial support.*** The MAL-ED study is carried out as a collaborative project supported by the Bill & Melinda Gates Foundation, the Foundation for the National Institutes of Health (NIH), and the NIH Fogarty International Center. This work was also supported by the National Institute of Allergy and Infectious Diseases of the NIH (grant numbers K23 AI108790 to P. K. and R01 AI043596 to W. A. P.) and by the Sherrilyn and Ken Fisher Center for Environmental Infectious Diseases Discovery Program (to P. D.).

***Potential conflicts of interest.*** A. L. reports a grant from Universidade Federal do Ceará. P. P. Y. reports an institutional grant from Johns Hopkins University. R. H. and A. S. G. F. report a grant from the University of Virginia/Bill & Melinda Gates Foundation. W. P. reports consultancy fees from TechLab, Inc. All other authors report no potential conflicts of interest. All authors have submitted the ICMJE Form for Disclosure of Potential Conflicts of Interest. Conflicts that the editors consider relevant to the content of the manuscript have been disclosed.

## References

[1] GBD Diarrhoeal Diseases Collaborators. Estimates of global, regional, and national morbidity, mortality, and aetiologies of diarrhoeal diseases: a systematic analysis for the Global Burden of Disease Study 2015. Lancet Infect Dis 2017; 17:909–48. 28579426 10.1016/S1473-3099(17)30276-1 PMC5589208

[2] Sow SO, Muhsen K, Nasrin D, et al The burden of *Cryptosporidium* diarrheal disease among children < 24 months of age in moderate/high mortality regions of sub-Saharan Africa and South Asia, utilizing data from the Global Enteric Multicenter Study (GEMS). PLoS Negl Trop Dis 2016; 10:e0004729. 27219054 10.1371/journal.pntd.0004729 PMC4878811

[3] Kotloff KL, Nataro JP, Blackwelder WC, et al Burden and aetiology of diarrhoeal disease in infants and young children in developing countries (the Global Enteric Multicenter Study, GEMS): a prospective, case-control study. Lancet 2013; 382:209–22. 23680352 10.1016/S0140-6736(13)60844-2

[4] Lima AA, Moore SR, Barboza MS Jr, et al Persistent diarrhea signals a critical period of increased diarrhea burdens and nutritional shortfalls: a prospective cohort study among children in northeastern Brazil. J Infect Dis 2000; 181:1643–51. 10823764 10.1086/315423

[5] Khan WA, Rogers KA, Karim MM, et al Cryptosporidiosis among Bangladeshi children with diarrhea: a prospective, matched, case-control study of clinical features, epidemiology and systemic antibody responses. Am J Trop Med Hyg 2004; 71:412–9. 15516636

[6] Bushen OY, Kohli A, Pinkerton RC, et al Heavy cryptosporidial infections in children in northeast Brazil: comparison of *Cryptosporidium hominis* and *Cryptosporidium parvum*. Trans R Soc Trop Med Hyg 2007; 101:378–84. 16934303 10.1016/j.trstmh.2006.06.005

[7] Checkley W, Gilman RH, Epstein LD, et al Asymptomatic and symptomatic cryptosporidiosis: their acute effect on weight gain in Peruvian children. Am J Epidemiol 1997; 145:156–63. 9006312 10.1093/oxfordjournals.aje.a009086

[8] Guerrant DI, Moore SR, Lima AA, Patrick PD, Schorling JB, Guerrant RL Association of early childhood diarrhea and cryptosporidiosis with impaired physical fitness and cognitive function four-seven years later in a poor urban community in northeast Brazil. Am J Trop Med Hyg 1999; 61:707–13. 10586898 10.4269/ajtmh.1999.61.707

[9] Korpe PS, Haque R, Gilchrist C, et al Natural history of cryptosporidiosis in a longitudinal study of slum-dwelling Bangladeshi children: association with severe malnutrition. PLoS Negl Trop Dis 2016; 10:e0004564. 27144404 10.1371/journal.pntd.0004564 PMC4856361

[10] Mondal D, Minak J, Alam M, et al Contribution of enteric infection, altered intestinal barrier function, and maternal malnutrition to infant malnutrition in Bangladesh. Clin Infect Dis 2012; 54:185–92. 22109945 10.1093/cid/cir807 PMC3245731

[11] Newman RD, Sears CL, Moore SR, et al Longitudinal study of *Cryptosporidium* infection in children in northeastern Brazil. J Infect Dis 1999; 180:167–75. 10353875 10.1086/314820

[12] Chacín-Bonilla L, Barrios F, Sanchez Y Environmental risk factors for *Cryptosporidium* infection in an island from western Venezuela. Mem Inst Oswaldo Cruz 2008; 103:45–9. 18345459 10.1590/s0074-02762008005000007

[13] Katsumata T, Hosea D, Wasito EB, et al Cryptosporidiosis in Indonesia: a hospital-based study and a community-based survey. Am J Trop Med Hyg 1998; 59:628–32. 9790442 10.4269/ajtmh.1998.59.628

[14] Solórzano-Santos F, Penagos-Paniagua M, Meneses-Esquivel R, et al *Cryptosporidium parvum* infection in malnourished and non malnourished children without diarrhea in a Mexican rural population [in Spanish]. Rev Invest Clin 2000; 52:625–31. 11256105

[15] Cruz JR, Cano F, Càceres P, Chew F, Pareja G Infection and diarrhea caused by *Cryptosporidium* sp. among Guatemalan infants. J Clin Microbiol 1988; 26:88–91. 3343318 10.1128/jcm.26.1.88-91.1988 PMC266196

[16] Mølback K, Andersen M, Aaby P, et al *Cryptosporidium* infection in infancy as a cause of malnutrition: a community study from Guinea-Bissau, West Africa. Am J Clin Nutr 1997; 65:149–52. 8988927 10.1093/ajcn/65.1.149

[17] Pavlinac PB, John-Stewart GC, Naulikha JM, et al High-risk enteric pathogens associated with HIV infection and HIV exposure in Kenyan children with acute diarrhoea. AIDS 2014; 28:2287–96. 25028987 10.1097/QAD.0000000000000396 PMC4346243

[18] Platts-Mills JA, Babji S, Bodhidatta L, Gratz J, Haque R, Havt A, et al Pathogen-specific burdens of community diarrhoea in developing countries: a multisite birth cohort study (MAL-ED). Lancet Glob Health 2015; 3:e564–75. 26202075 10.1016/S2214-109X(15)00151-5 PMC7328884

[19] Ahmed T, Mahfuz M, Islam MM, et al The MAL-ED cohort study in Mirpur, Bangladesh. Clin Infect Dis 2014; 59(Suppl 4):S280–6. 25305298 10.1093/cid/ciu458

[20] Lima AA, Oria RB, Soares AM, et al Geography, population, demography, socioeconomic, anthropometry, and environmental status in the MAL-ED cohort and case-control study sites in Fortaleza, Ceara, Brazil. Clin Infect Dis 2014; 59(Suppl 4):S287–94. 25305299 10.1093/cid/ciu438

[21] John SM, Thomas RJ, Kaki S, Sharma SL, Ramanujam K, Raghava MV, et al Establishment of the MAL-ED birth cohort study site in Vellore, Southern India. Clin Infect Dis 2014; 59(Suppl 4):S295–9. 25305300 10.1093/cid/ciu390

[22] Shrestha PS, Shrestha SK, Bodhidatta L, Strand T, Shrestha B, Shrestha R, et al Bhaktapur, Nepal: the MAL-ED birth cohort study in Nepal. Clin Infect Dis 2014; 59(Suppl 4):S300–3. 25305301 10.1093/cid/ciu459

[23] Yori PP, Lee G, Olortegui MP, Chavez CB, Flores JT, Vasquez AO, et al Santa Clara de Nanay: the MAL-ED cohort in Peru. Clin Infect Dis 2014; 59(Suppl 4):S310–6. 25305303 10.1093/cid/ciu460

[24] Turab A, Soofi SB, Ahmed I, Bhatti Z, Zaidi AK, Bhutta ZA Demographic, socioeconomic, and health characteristics of the MAL-ED network study site in rural Pakistan. Clin Infect Dis 2014; 59(Suppl 4):S304–9. 25305302 10.1093/cid/ciu391

[25] Bessong PO, Nyathi E, Mahopo TC, Netshandama V; MAL-ED South Africa Development of the Dzimauli community in Vhembe District, Limpopo province of South Africa, for the MAL-ED cohort study. Clin Infect Dis 2014; 59(Suppl 4):S317–24. 25305304 10.1093/cid/ciu418

[26] Mduma ER, Gratz J, Patil C, et al The etiology, risk factors, and interactions of enteric infections and malnutrition and the consequences for child health and development study (MAL-ED): description of the Tanzanian site. Clin Infect Dis 2014; 59(Suppl 4):S325–30. 25305305 10.1093/cid/ciu439

[27] Richard SA, Barrett LJ, Guerrant RL, Checkley W, Miller MA; MAL-ED Network Investigators Disease surveillance methods used in the 8-site MAL-ED cohort study. Clin Infect Dis 2014; 59(Suppl 4):S220–4. 25305290 10.1093/cid/ciu435 PMC4204606

[28] Houpt E, Gratz J, Kosek M, Zaidi AK, Qureshi S, Kang G, et al Microbiologic methods utilized in the MAL-ED cohort study. Clin Infect Dis 2014; 59(Suppl 4):S225–32. 25305291 10.1093/cid/ciu413 PMC4204609

[29] United Nations. Millennium Development Goals indicators 2008 Available at: http://mdgs.un.org/unsd/mdg/Metadata.aspx?IndicatorId=0&SeriesId=711. Accessed 1 May 2018.

[30] World Health Organization/United Nations Children’s Fund Joint Monitoring Programme for Water Supply, Sanitation and Hygiene. Improved and unimproved water sources and sanitation facilities 2015 Available at: http://www.wssinfo.org/definitions-methods/watsan-categories/. Accessed 1 May 2018.

[31] Psaki SR, Seidman JC, Miller M, et al Measuring socioeconomic status in multicountry studies: results from the eight-country MAL-ED study. Popul Health Metr 2014; 12:8. 24656134 10.1186/1478-7954-12-8 PMC4234146

[32] Kotloff KL, Blackwelder WC, Nasrin D, et al The Global Enteric Multicenter Study (GEMS) of diarrheal disease in infants and young children in developing countries: epidemiologic and clinical methods of the case/control study. Clin Infect Dis 2012; 55(Suppl 4):S232–45. 23169936 10.1093/cid/cis753 PMC3502307

[33] Korpe PS, Liu Y, Siddique A, et al Breast milk parasite-specific antibodies and protection from amebiasis and cryptosporidiosis in Bangladeshi infants: a prospective cohort study. Clin Infect Dis 2013; 56:988–92. 23243179 10.1093/cid/cis1044 PMC3588117

[34] Kattula D, Jeyavelu N, Prabhakaran AD, et al Natural history of cryptosporidiosis in a birth cohort in southern India. Clin Infect Dis 2017; 64:347–54. 28013266 10.1093/cid/ciw730 PMC5241779

[35] Liu J, Platts-Mills JA, Juma J, et al Use of quantitative molecular diagnostic methods to identify causes of diarrhoea in children: a reanalysis of the GEMS case-control study. Lancet 2016; 388:1291–301. 27673470 10.1016/S0140-6736(16)31529-X PMC5471845

[36] Sarkar R, Ajjampur SS, Prabakaran AD, et al Cryptosporidiosis among children in an endemic semiurban community in southern India: does a protected drinking water source decrease infection?Clin Infect Dis 2013; 57:398–406. 23709650 10.1093/cid/cit288 PMC3703109

[37] Cama VA, Bern C, Roberts J, et al *Cryptosporidium* species and subtypes and clinical manifestations in children, Peru. Emerg Infect Dis 2008; 14:1567–74. 18826821 10.3201/eid1410.071273 PMC2609889

[38] Ajjampur SS, Gladstone BP, Selvapandian D, Muliyil JP, Ward H, Kang G Molecular and spatial epidemiology of cryptosporidiosis in children in a semiurban community in south India. J Clin Microbiol 2007; 45:915–20. 17251402 10.1128/JCM.01590-06 PMC1829120

[39] Carmolli M, Duggal P, Haque R, et al Deficient serum mannose-binding lectin levels and MBL2 polymorphisms increase the risk of single and recurrent *Cryptosporidium* infections in young children. J Infect Dis 2009; 200:1540–7. 19827946 10.1086/606013 PMC3400050

