# Supplementary material for: Epidemiology and Risk Factors for Cryptosporidiosis in Children From 8 Low-income Sites: Results From the MAL-ED Study
Source: Clin Infect Dis. 2018 Apr 26;67(11):1660–9. doi: 10.1093/cid/ciy355 (PMC6233690; doi:10.1093/cid/ciy355)
Supplement: Supplemental_Table_1 [file ciy355_suppl_supplemental_table_1.docx]

**Supplemental Table 1**. Site-specific Multivariate Logistic Regression of risk factors for *Cryptosporidium* infection during the first 24 months of life.

| Risk Factors |  | **Odds Ratio (95% C.I.)** | | | | | |  |
| --- | --- | --- | --- | --- | --- | --- | --- | --- |
|  | **BGD** | **INV** | **NEB** | **PKN** | **PEL** | **SAV** | **TZH** | |
| Overcrowding | 2.33 (1.18, 4.61) | 1.24 (0.69, 2.26) | 1.21 (0.45, 3.21) | 0.95 (0.50, 1.80) | 1.03 (0.28, 3.80) | 1.72 (0.49, 6.14) | 0.84 (0.27, 2.62) | |
| Dirt Floor | 1.66 (0.41, 6.74) | 0.80 (0.27, 2.38) | 0.99 (0.53, 1.85) | 1.65 (0.78, 3.46) | 1.72 (0.71, 4.13) | * | * | |
| Poor Sanitation | * | * | * | 1.21 (0.55, 2.68) | 0.87 (0.34, 19.85) | * | * | |
| Unprotected water source | * | * | * | * | 2.49 (0.31, 19.84) | 1.86 (0.59, 5.83) | 0.98 (0.40, 2.43) | |
| Chickens or ducks kept in home | 5.26 (0.64, 42.89) | 0.91 (0.34, 2.43) | 1.52 (0.80, 2.87) | 0.65 (0.34, 1.23) | 1.15 (0.51, 2.61) | 0.86 (0.40, 1.86) | * | |
| Cattle kept in home | * | * | * | 0.85 (0.44, 1.67) | * | 0.43 (0.14, 1.32) | 0.83 (0.32, 2.17) | |
| Maternal Schooling 1-5 years | 0.83 (0.35, 1.98) | 0.61 (0.22, 1.71) | 0.87 (0.25, 2.97) | 0.84 (0.43, 1.65) | * | * | 0.67 (0.19, 2.38) | |
| Maternal Schooling > 5 years | 1.95 (0.78, 4.87) | 0.51 (0.20, 1.31) | 0.61 (0.20, 1.87) | 0.77 (0.33, 1.83) | * | * | 1.41 (0.46, 4.29) | |
| Household income (log) | 0.96  (0.53, 1.74) | 1.65  (0.98, 2.76) | 0.90  (0.55, 1.48) | 1.15  (0.72, 1.84) | 1.30  (0.77, 2.17) | 0.94  (0.07, 15.0) | 1.89  (1.14, 3.12) | |

*Only variables with OR’s listed were included in the model in each site. Variables with less than 5% heterogeneity between categories were not included due to lack of power.
