# Supplementary material for: Epidemiology and Risk Factors for Cryptosporidiosis in Children From 8 Low-income Sites: Results From the MAL-ED Study
Source: Clin Infect Dis. 2018 Apr 26;67(11):1660–9. doi: 10.1093/cid/ciy355 (PMC6233690; doi:10.1093/cid/ciy355)
Supplement: Supplemental_Table_2 [file ciy355_suppl_supplemental_table_2.docx]

**Supplemental Table 2.** LAZ as predictor of *Cryptosporidium* infection. The mean 3-month LAZ prior to time of infection was compared between infected and uninfected children. In ages 9- and 12-months, lower preceding LAZ was associated with *Cryptosporidium* infection.

| Age at time of infection | Mean LAZ over prior 3 months | | *p*-value |
| --- | --- | --- | --- |
|  | Crypto negative (*%*) | Crypto positive (*%*) |  |
| 3 months (n = 1724) | - 0.97 (96.0) | - 0.88 (4.0) | 0.57 |
| 6 months (n = 1702) | - 1.0 (96.5) | -1.2 (3.5) | 0.06 |
| 9 months (n = 1685) | -1.1 (96.7) | -1.3 (4.3) | 0.05 |
| 12 months (n = 1662) | -1.3 (95.3) | -1.6 (4.7) | 0.007 |

* Welch’s two-sample T-test
