# Supplementary material for: Epidemiology and Risk Factors for Cryptosporidiosis in Children From 8 Low-income Sites: Results From the MAL-ED Study
Source: Clin Infect Dis. 2018 Apr 26;67(11):1660–9. doi: 10.1093/cid/ciy355 (PMC6233690; doi:10.1093/cid/ciy355)
Supplement: Supplemental_Table_3 [file ciy355_suppl_supplemental_table_3.docx]

**Supplemental Table 3.**

Linear regression of association of *Cryptosporidium* infection (includes both diarrheal and subclinical) in the first 12 months of life and LAZ at 12 and 18 months. Total number of children included was 1328 (a total of 222 children from Pakistan were excluded).

|  | **Beta (95% CI)** | |
| --- | --- | --- |
| Site | **12-month LAZ** | **18-month LAZ** |
| BGD | -0.05 (-0.29, 0.18) | -0.21 (-0.45, 0.03) |
| BRF | 0.26 (-0.19, 0.70) | -0.07 (-0.56, 0.42) |
| INV | -0.25 (-0.47, -0.03) | -0.22 (-0.45, 0.02) |
| NPL | 0.07 (-0.17, 0.31) | -0.01 (-0.27, 0.25) |
| PEL | 0.01 (-0.18, 0.21) | -0.03 (-0.24, 0.19) |
| SAV | 0.19 (-0.19, 0.56) | 0.08 (-0.31, 0.46) |
| TZH | 0.25 (-0.01, 0.51) | -0.21 (-0.45, 0.03) |
