# Supplementary figures and images for: Epidemiology and Risk Factors for Cryptosporidiosis in Children From 8 Low-income Sites: Results From the MAL-ED Study
Source: Clin Infect Dis. 2018 Apr 26;67(11):1660–9. doi: 10.1093/cid/ciy355 (PMC6233690; doi:10.1093/cid/ciy355)

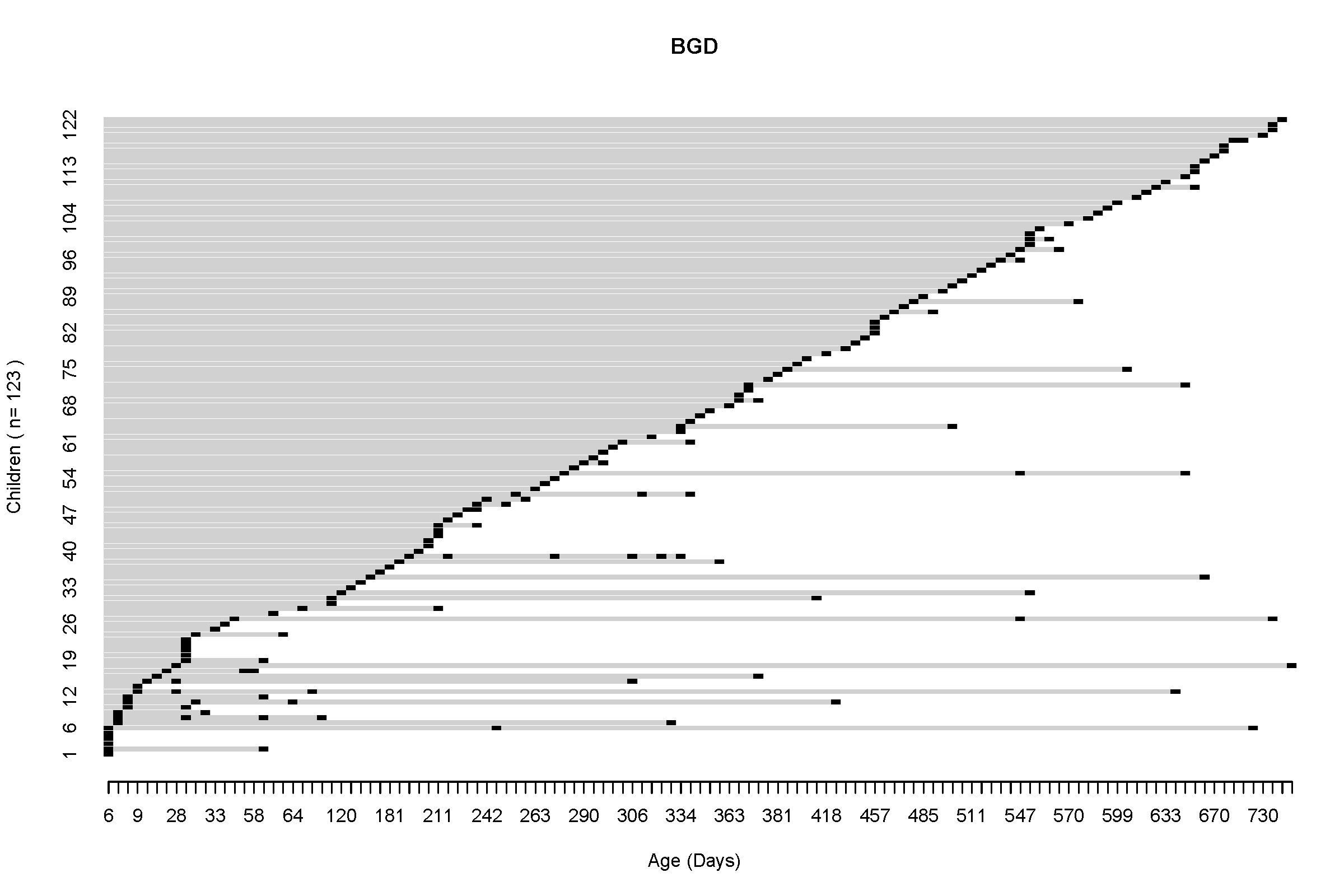

Supplement: Supplemental_Figure_1_Page_1 [file ciy355_suppl_supplemental_figure_1_page_1.png]

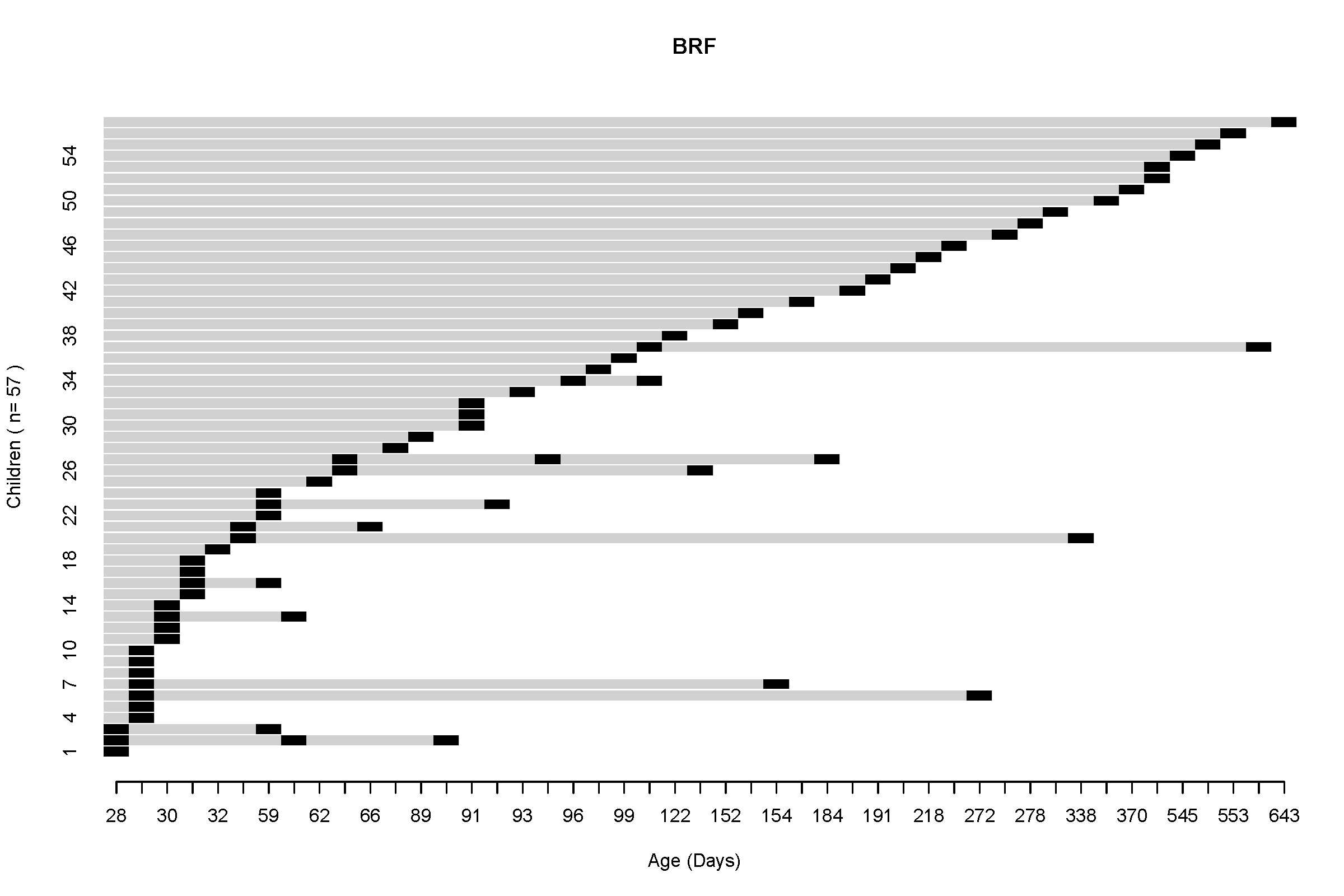

Supplement: Supplemental_Figure_1_Page_2 [file ciy355_suppl_supplemental_figure_1_page_2.png]

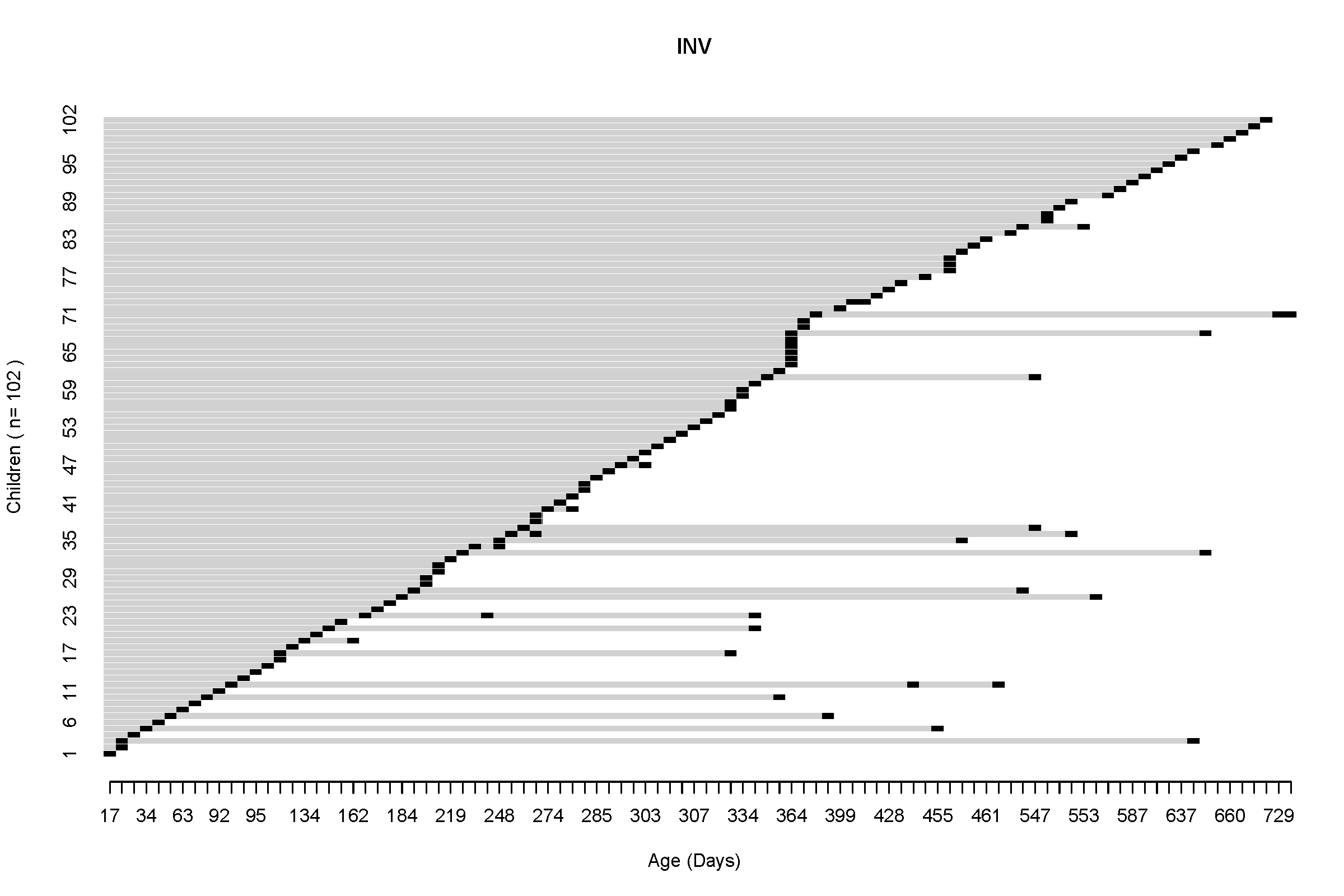

Supplement: Supplemental_Figure_1_Page_3 [file ciy355_suppl_supplemental_figure_1_page_3.png]

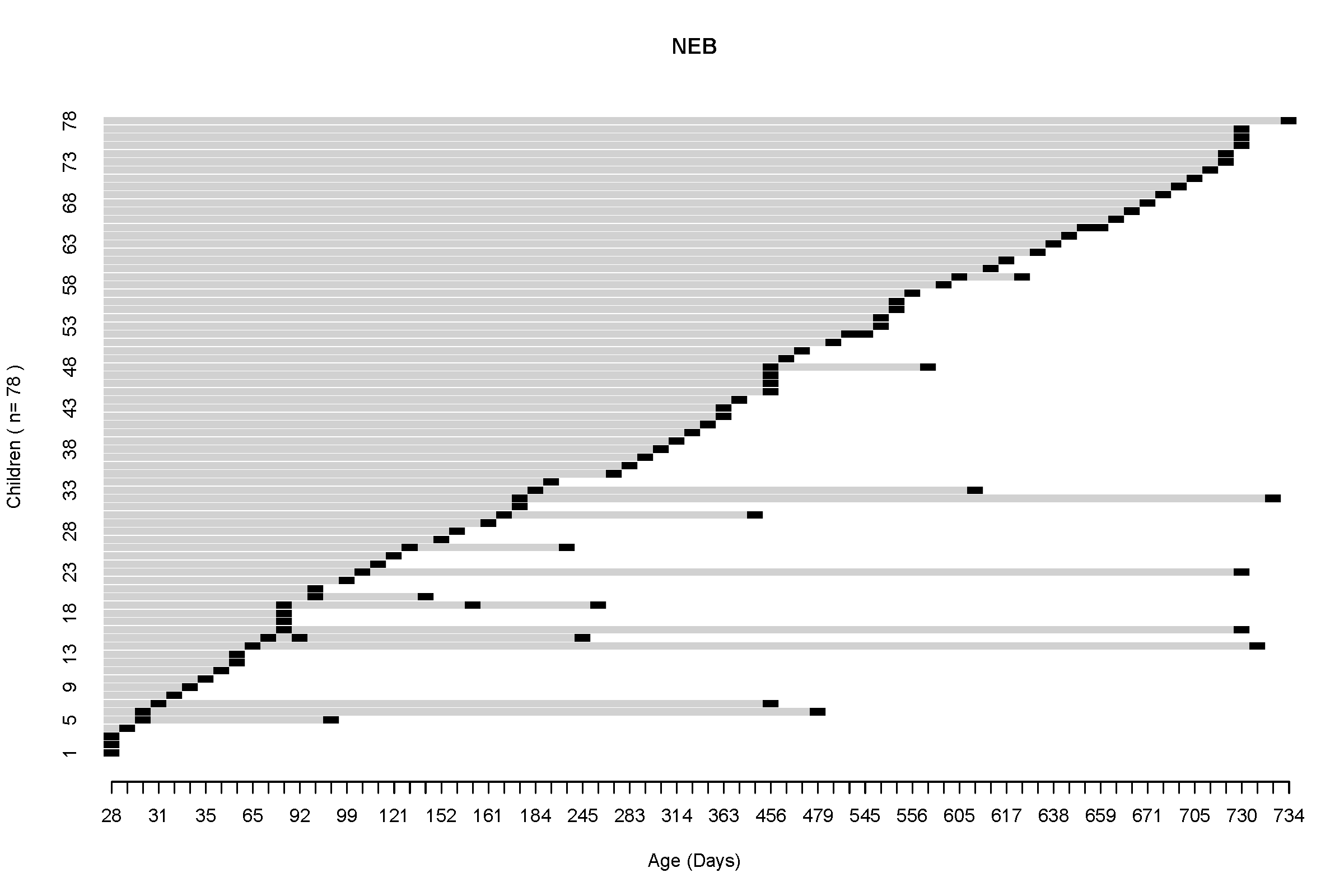

Supplement: Supplemental_Figure_1_Page_4 [file ciy355_suppl_supplemental_figure_1_page_4.png]

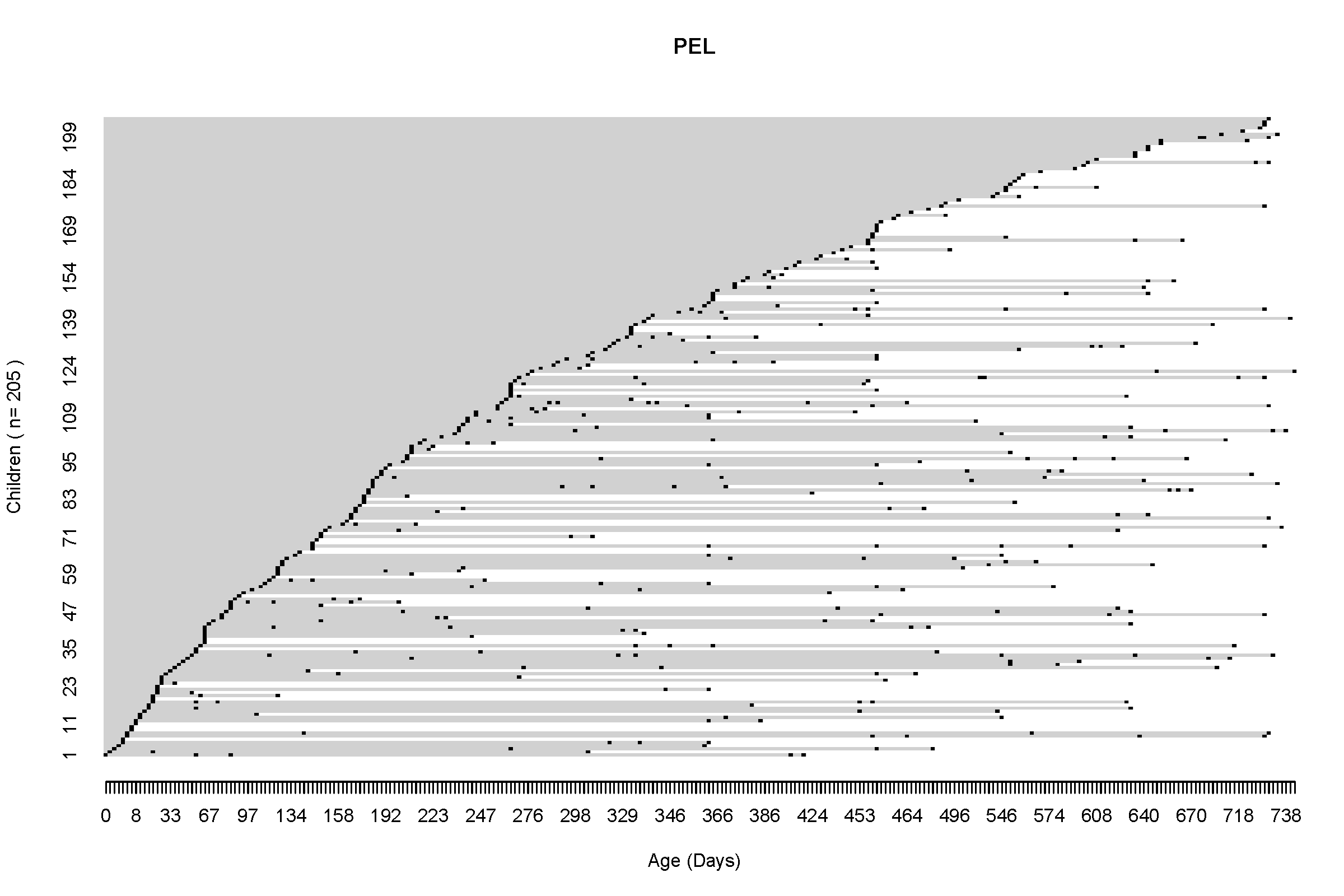

Supplement: Supplemental_Figure_1_Page_5 [file ciy355_suppl_supplemental_figure_1_page_5.png]

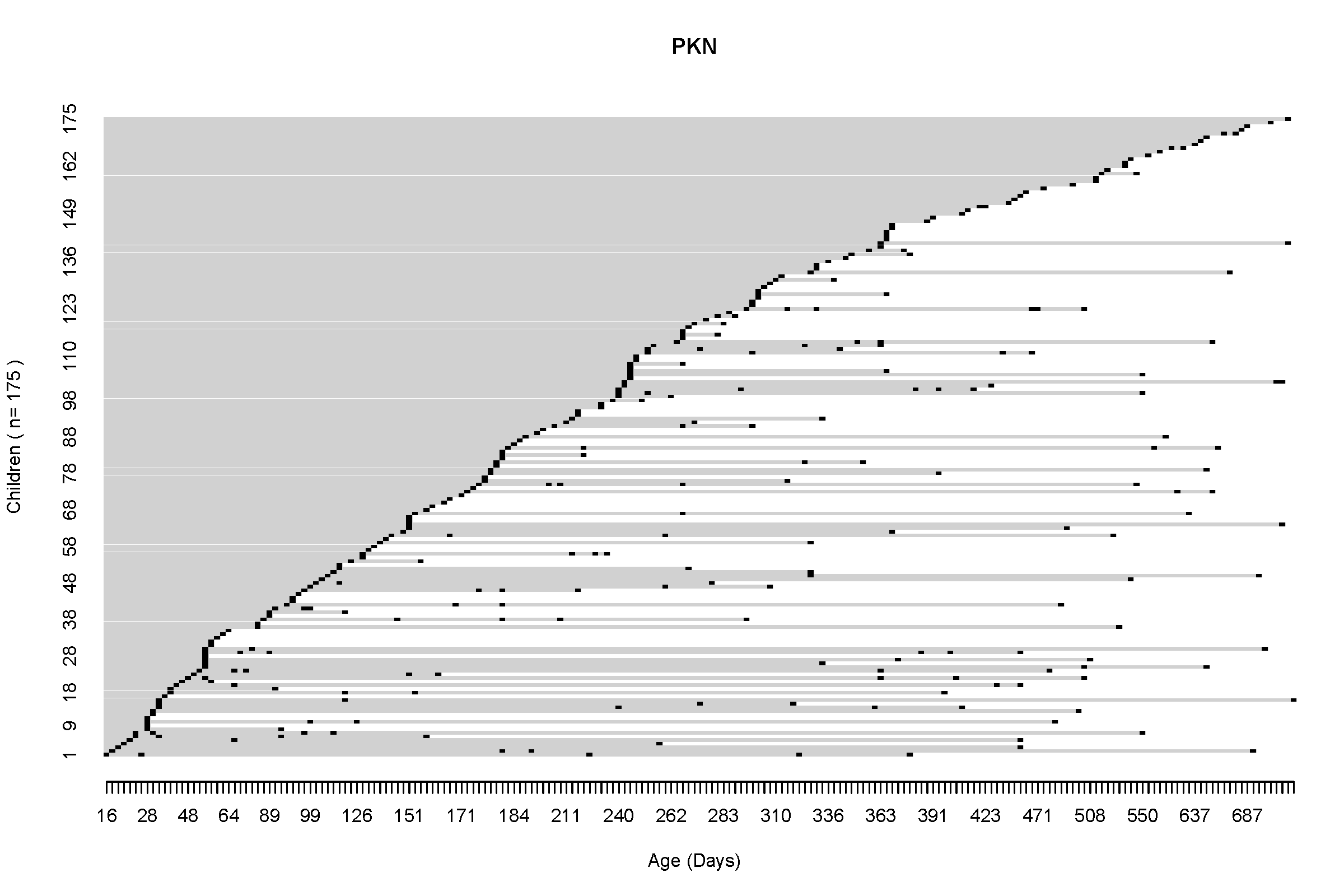

Supplement: Supplemental_Figure_1_Page_6 [file ciy355_suppl_supplemental_figure_1_page_6.png]

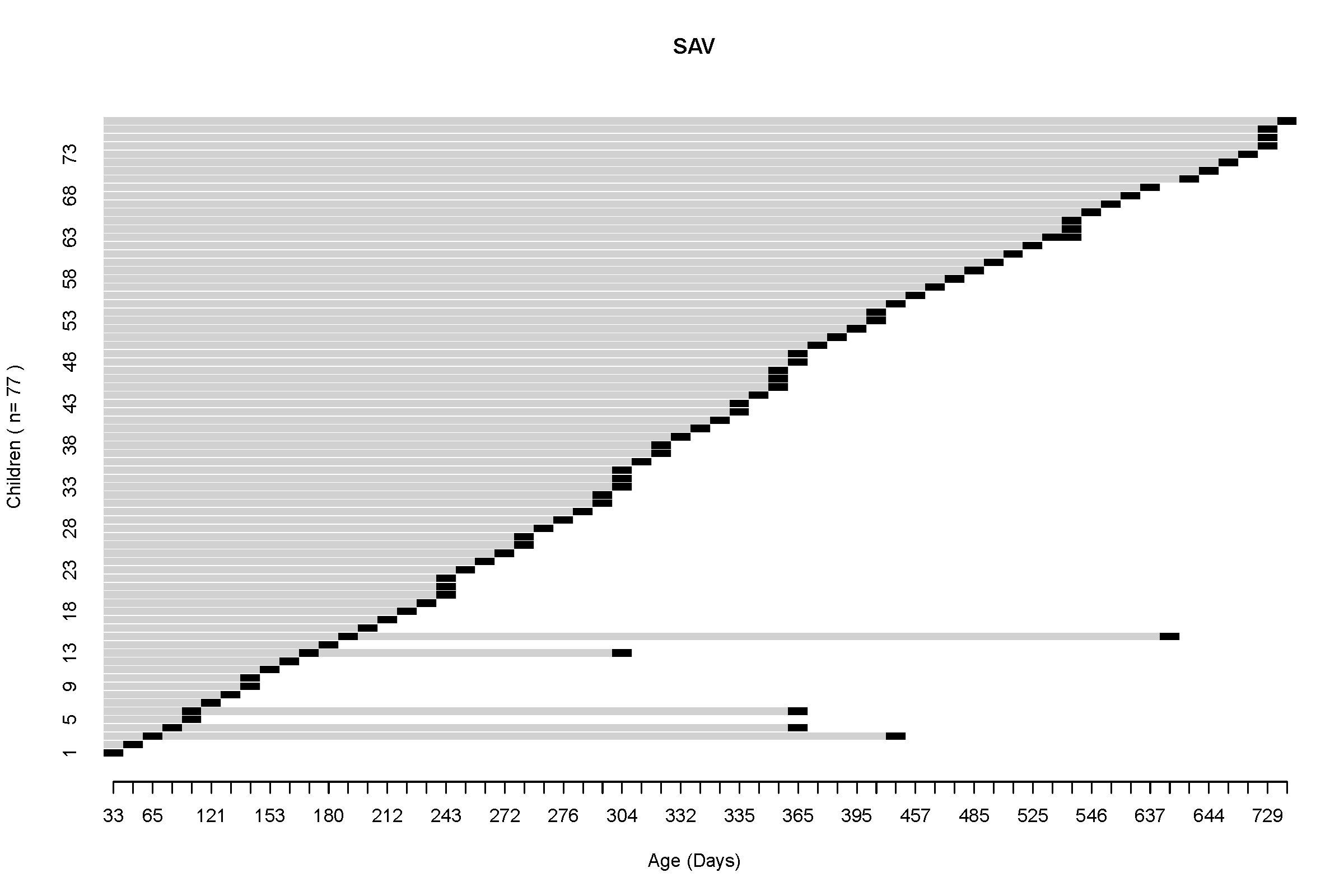

Supplement: Supplemental_Figure_1_Page_7 [file ciy355_suppl_supplemental_figure_1_page_7.png]

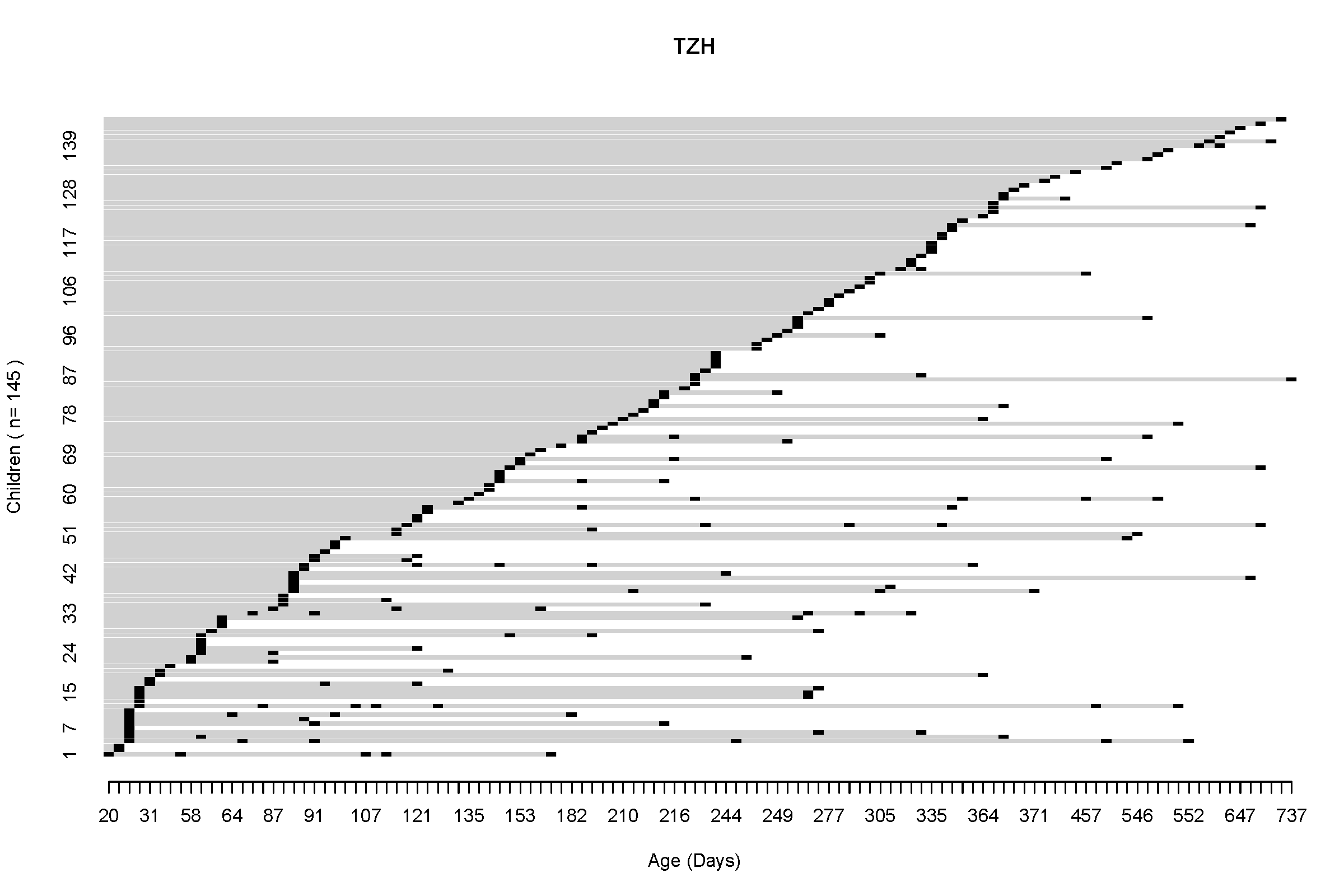

Supplement: Supplemental_Figure_1_Page_8 [file ciy355_suppl_supplemental_figure_1_page_8.png]
