# Supplementary material for: Epidemiology and Risk Factors for Cryptosporidiosis in Children From 8 Low-income Sites: Results From the MAL-ED Study
Source: Clin Infect Dis. 2018 Apr 26;67(11):1660–9. doi: 10.1093/cid/ciy355 (PMC6233690; doi:10.1093/cid/ciy355)
Supplement: Supplemental_Figure_legend [file ciy355_suppl_supplemental_figure_legend.docx]

**Supplemental Figure 1. Sequence Index Plot of *Cryptosporidium* Shedding by Site.**

Each child is represented by a row on the x-axis. The Y-axis represents age in days. Each protocol collected monthly stool and diarrheal stool is depicted per child row. Dark shading indicates a time point when the child tested positive for *Cryptosporidium spp,* light shading indicates the stool sample tested negative. Recurrent shedding is common in PEL, PKN, and TZH.
